# Pesquisa e cuidado à saúde no Instituto Fernandes Figueira: depoimento de José Augusto de Britto

**DOI:** 10.1590/S0104-59702024000100004

**Published:** 2024-04-05

**Authors:** José Augusto de Britto, Luiz Antonio Teixeira, Larissa Velasquez de Souza

**Affiliations:** i Médico pediatra, Instituto Nacional de Saúde da Mulher, da Criança e do Adolescente Fernandes Figueira/Fiocruz. Rio de Janeiro – RJ – Brasil de.britto@uol.com.br; ii Coordenador adjunto, Programa de Pós-graduação em Saúde da Família/Universidade Estácio de Sá; professor, Programa de Pós-graduação em Saúde da Criança e da Mulher/Instituto Nacional de Saúde da Mulher, da Criança e do Adolescente Fernandes Figueira/Fiocruz. Rio de Janeiro – RJ – Brasil luiz.teixeira@fiocruz.br; iii Pós-doutoranda, Programa de Pós-graduação em Saúde da Criança e da Mulher/ Instituto Nacional de Saúde da Mulher, da Criança e do Adolescente Fernandes Figueira/Fiocruz. Rio de Janeiro – RJ – Brasil larissasouzavelasquez@gmail.com

**Keywords:** History, Memory, Fernandes Figueira Institute, História, Memória, Instituto Fernandes Figueira

## Abstract

O depoimento resulta de entrevista realizada com José Augusto Alves de Britto, médico pediatra que ocupou o cargo de diretor do Instituto Fernandes Figueira entre 2001 e 2008. O relato do depoente aborda diferentes aspectos da história do instituto, como o desenvolvimento da pesquisa e do cuidado à saúde e transformações no cotidiano institucional. O depoimento faz parte de um projeto que documenta e investiga a história do Instituto Fernandes Figueira, que completa seu centenário em 2024.

## Abstract (en)

This material is the result of an interview with José Augusto Alves de Britto, a physician who served as director of the Fernandes Figueira Institute from 2001 to 2008. It covers different aspects of the history of the institution, such as research development, health care, and transformations in the daily routine there. It is part of a project to document and investigate the history of the Fernandes Figueira Institute, which celebrates its one hundredth anniversary in 2024.

A história institucional é perpassada por trajetórias individuais e coletivas que estabelecem práticas, interações, espaços e valores ao longo do tempo. Enquanto as experiências de algumas pessoas se vinculam às instituições de forma mais pontual ou tangencial, outras ganham contornos entrelaçados e duradouros, levando a certa imprecisão entre o que é trajetória do indivíduo ou do grupo e o que se refere à instituição em questão. O depoimento a seguir, certamente, enquadra-se na segunda categoria. José Augusto Alves de Britto é médico pediatra e trabalha no Instituto Nacional de Saúde da Mulher, da Criança e do Adolescente Fernandes Figueira (IFF) desde 1980, tendo ocupado diferentes posições na instituição, inclusive a de diretor entre 2001 e 2008. Também possui especialização em nutrologia pela Universidade Federal do Estado do Rio de Janeiro, em gestão hospitalar pela Escola Nacional de Saúde Pública Sergio Arouca, e mestrado e doutorado em pediatria pelo Instituto Fernandes Figueira.

O depoimento de José Augusto aborda diferentes aspectos da história do IFF pela perspectiva de um ator com posição política relevante em termos institucionais, o que ajusta sua lente para pontos específicos. São abordados elementos que, na perspectiva do depoente, marcaram o processo de estruturação do instituto e o tornaram modernizado e com reconhecimento dentro da Fundação Oswaldo Cruz (Fiocruz) e em âmbitos nacional e internacional. Também compõem este relato traços do cotidiano institucional, como a relação com a sociedade civil, dinâmicas profissionais internas e a caracterização do IFF como hospital e instituto.

Este depoimento é resultado de uma entrevista realizada por Luiz Antonio Teixeira e Larissa Velasquez de Souza com José Augusto de Britto, parte de um projeto que documenta e investiga a história do Instituto Fernandes Figueira, que completa seu centenário em 2024. A entrevista foi editada e transformada em depoimento, seguindo ordem temática e cronológica do que foi relatado pelo depoente, que se encontrava no próprio instituto durante a gravação, explicando, assim, o uso de “aqui” para se referir ao IFF em momentos diversos do texto.

## Depoimento de José Augusto Alves de Britto

Minha trajetória no Instituto Fernandes Figueira começa bem antes do meu período como diretor, entre 2001 e 2008. Eu entrei em 1980, poucos anos depois de o instituto ser incorporado à Fiocruz, em 1978. O IFF possui longa história de atuação nas políticas de saúde para a infância no país, sendo vinculado ao Departamento Nacional da Criança. O Fernandes Figueira, médico que atuou aqui e dá nome ao instituto, foi uma pessoa muito relevante, ele mudou o jeito de cuidar da criança, atuando na humanização da relação entre mãe e filho. A trajetória do Fernandes Figueira também desenhou um perfil da instituição na área da pediatria, um dos seus carros-chefe. Quando ingressei, havia profissionais de destaque na área, como Fernando Olinto,^1^ que dirigia um centro de estudos bastante famoso no Rio de Janeiro, para onde ele trazia todas as celebridades da medicina do Brasil. Eu tive aula com todos os grandes nomes da pediatria do Brasil! Nessa época, as áreas de ginecologia, obstetrícia e cirurgia pediátrica também possuíam grande reconhecimento, com nomes como Clóvis Corrêa da Costa,^2^ que dá nome à maternidade do instituto. Essas áreas eram compostas por profissionais com experiência na instituição, que já eram antigos no IFF quando entrei aqui; então, eram grupos já consolidados.

Das muitas áreas de atuação do Instituto Fernandes Figueira, algumas foram fundamentais na trajetória institucional, tendo caráter estruturante e ressaltando nossa missão de cuidado materno-infantil. Eu destacaria a neonatologia, a criação e funcionamento do banco de leite,^3^ a maternidade, hoje referência para gestações de risco fetal, e o serviço de genética. Houve também grupos e pessoas importantes que atuaram em áreas mais específicas e inéditas no Brasil, como a doutora Ludma Dallalana Trota,^4^ referência em pesquisas sobre a fibrose cística, importante doença rara. A Ludma foi a primeira pessoa a diagnosticar um caso da doença no Brasil, e coordenava uma enfermaria específica aqui no IFF. Do mesmo jeito, existia uma enfermaria da pneumologia que só lidava com casos de tuberculose infantil, na época em que ainda se realizavam abreugrafias^5^ aqui no hospital.

É interessante observar como o crescimento dessas diferentes áreas no instituto criaram formas de reconhecimento variadas entre a população. Durante muito tempo, o Fernandes Figueira foi “o berçário”, por conta da neonatologia; se você entra em um táxi hoje e diz que está vindo para cá, pode ouvir algo do tipo “ah, aquela maternidade que tem ali na avenida Rui Barbosa”, ou “ah, é o banco de leite”. Essas formas de identificar a instituição foram mudando também ao longo do tempo, à medida que as áreas foram se desenvolvendo. Esse processo de transformação do hospital ocorreu tanto por dinâmicas internas quanto por outras ligadas ao contexto político do país, da saúde pública e da Fiocruz em um sentido mais amplo.

Na década de 1980, durante a presidência do Sergio Arouca na Fiocruz^6^ e a diretoria do Paulo Roberto Boechat^7^ aqui no IFF, ocorreram mudanças importantes na dinâmica institucional, a exemplo da relação entre os departamentos, a abertura de agendas de pesquisa em campos mais relacionados ao cenário da saúde pública, da medicina social e da saúde coletiva e, em termos políticos, mais visibilidade do Instituto Fernandes Figueira no quadro geral da Fundação Oswaldo Cruz. Para mim, o período de gestão do Arouca e do Boechat foi um sopro de modernidade para o hospital, possibilitando mudanças que foram aprofundadas nos anos posteriores, inclusive no período em que fui diretor do instituto. Estávamos saindo de um período de governo militar, em que aspectos da gestão eram influenciados pela lógica hierárquica de comando. No período de Arouca e Boechat, essa hierarquia foi rompida, como parte de um projeto de socialização das interações dentro da instituição, uma tentativa de acabar com os “feudos” que existiam no IFF, no sentido de isolamento dos departamentos.

Outro personagem importante nessa dimensão da política institucional foi Paulo Buss,^8^ que presidiu a Fiocruz entre 2000 e 2008, o que inclui todo o período em que dirigi o IFF. Sua gestão foi fundamental para nós; ele reavivou o hospital, trazendo investimentos e força política para a instituição que, alinhada às políticas da saúde da mulher, da criança e do adolescente, recebeu a visita de ministros da Saúde. Um ponto de grande importância durante nossa gestão, a dele na presidência e a minha no instituto, foi a realização de um concurso público em 2000, com duzentas vagas destinadas aos quadros do IFF, transformando a instituição, tanto na dimensão assistencial quanto na pesquisa e no ensino – o tripé da missão institucional da Fiocruz.

No âmbito da pesquisa e do ensino, mudanças relevantes vieram com a criação da pós-graduação, em 1988, embora sempre houvesse um discurso de que o IFF fosse “um hospital que fazia pesquisas”. Quando ingressei no IFF, aliás, eu o escolhi porque aqui se fazia pesquisa. Além disso, já havia, antes dos mestrados e doutorados, uma tradicional residência em pediatria, referência no Rio de Janeiro e responsável por formar profissionais de diversos estados da federação que, como egressos, tiveram notável atuação nas políticas públicas de saúde, assumindo secretarias de saúde e serviços hospitalares nos campos da pediatria, da neonatologia, da obstetrícia, principalmente na região Nordeste do país. Essa tradição na residência era algo muito forte no instituto, mas o desenvolvimento da pós-graduação teve grande impacto na forma como a pesquisa passou a ser feita aqui dentro. Certa vez, eu disse ao Paulo Buss, então presidente da Fiocruz, que “a gente vive fazendo pesquisa, mas é pesquisa como pesquisador individual, não é pesquisa institucional”, e era esse mesmo o cenário que tínhamos. Havia importantes pesquisadores atuando no IFF, mas de forma mais isolada, sem estrutura e planejamento institucional para desenvolvimento dos seus projetos e consolidação dos seus grupos de pesquisa. Com a criação dos mestrados e doutorados e os investimentos feitos pelo governo federal durante o primeiro e o segundo governos Lula, tivemos a possibilidade de fortalecer a pesquisa e o ensino no instituto.

Também durante a minha gestão nos envolvemos em importantes ações de cooperação internacional, a exemplo da estabelecida com os países africanos de língua oficial portuguesa. Fizemos muitos trabalhos no âmbito da saúde materno-infantil na África e na Ásia, para onde eu mesmo fui duas vezes, em viagens de ações de cooperação. Chegando lá, vi situações assim: como a mesa de parto estava quebrada, deitaram a mulher no chão, fizeram o parto dela no chão, retiraram a mulher do chão, veio uma profissional limpando e secando com os pés o sangue do chão. Isso em um hospital na África, onde cerca de 30% das gestantes tinham sorologia positiva para o HIV. Nós interferimos nisso, promovendo humanização das práticas, alterando posições das camas, dialogando com curandeiros das tribos e médicos dos serviços locais. Foram esforços diversos, enviando profissionais da enfermagem obstétrica, da pediatria, da obstetrícia, entre outros. Houve mesmo a proposta de criação de serviços de banco de leite em Moçambique e Angola, além de ações no Timor Leste e em Cabo Verde, com envio de funcionários do IFF que se estabeleceram nos territórios por um período mais longo. Infelizmente, com as mudanças no ambiente político e nas diretrizes ministeriais, essas ações de cooperação foram gradualmente abandonadas ou enfraquecidas. Tivemos também ações em outros países, como Escócia, França, Espanha e Inglaterra. Mas eu destaco esse movimento de aproximação dos países africanos, foi um momento lindo.

Outro elemento importante na estruturação do instituto foram os grupos e iniciativas no âmbito da sociedade civil, como o Instituto Refazer,^9^ que abraça crianças com necessidades especiais e auxilia no cuidado a elas aqui no IFF, alugando equipamentos respiradores, por exemplo. Eram organizados eventos beneficentes reunindo pessoas abonadas, arrecadando recursos para apoiar com equipamentos as pesquisas do hospital. Foi assim que conseguimos a criação de uma academia de musculação para pacientes com condições incapacitantes como a fibrose cística. Todos os anos eram realizados jantares no Copacabana Palace pela embaixatriz Naná Sette Câmara, que sempre destinava parte do que arrecadava para a gente. Foi ela quem assinou o cheque com o dinheiro necessário para criarmos a academia com equipamentos diferenciados, próprios para as crianças. Foi genial!

Ainda na dimensão da assistência social, tivemos uma parceria muito grande com a França, tão boa que a Carla Bruni, na época primeira-dama da França, veio visitar o hospital, em uma ação organizada com a Embaixada da França no Brasil para a doação de dois carros da Renault para o IFF, dois utilitários. Esses veículos eram utilizados pelo Programa de Assistência Domiciliar, destinado ao atendimento de casos crônicos e complexos. Assim, além de as crianças serem levadas de volta para casa depois de internações, também se deslocavam até lá profissionais de medicina, enfermagem, e nutricionistas para visitá-las. Esse programa, por sinal, enquadra-se, entre as transformações que o hospital passou devido à própria mudança no perfil epidemiológico de nossos pacientes. Cada vez mais recebíamos crianças em quadros crônicos e complexos, e isso impactou na rotina hospitalar. Por exemplo, não era permitido às mães acompanhar seus filhos mais de perto, pois essa era a dinâmica na época.

Em todas essas frentes, fomos nos consolidando como um instituto. Acompanhei essa discussão ao longo dos anos, com puristas dizendo que somos um instituto que possui um hospital com linhas de pesquisa, de formação e de assistência. Essas tensões na definição se devem, em parte, aos recursos. O Instituto Fernandes Figueira tinha um dos maiores orçamentos dentro da Fiocruz, o que gerava tensões no momento de votação desses. E o questionamento era exatamente na linha de sermos um hospital, ao que eu respondia “não somos só um hospital. Somos um instituto que tem um hospital”. Essa disputa mudou um pouco com a transformação do IFF e do Instituto Evandro Chagas em institutos nacionais, passando a propor políticas nacionais em suas áreas e assumindo um papel mais próximo à saúde pública. A questão do hospital ou instituto também afetava certos aspectos profissionais, principalmente na definição do profissional como médico ou pesquisador, pois isso implicava o ponto da dedicação exclusiva e, por consequência, o salário das pessoas. Foi também durante a gestão de Paulo Buss como presidente da Fiocruz que fizemos uma intensa mobilização para equiparar os salários de médicos e pesquisadores aqui no instituto.

Houve, ainda, aspectos específicos da trajetória institucional que tiveram muito êxito e foram importantes na estruturação do IFF como conhecemos hoje. Na área da genética clínica, tivemos o desenvolvimento de pesquisas relevantes e a transformação do instituto em centro de referência no cuidado a portadores de doenças genéticas raras, a partir das políticas nacionais de genética clínica e de doenças raras, de 2009 e 2014.^10^ Nesse campo, tivemos articulações fundamentais com o setor privado e os movimentos sociais, principalmente para a obtenção de medicamentos com custo elevado que envolviam, muitas vezes, a judicialização para garantia do acesso. O ponto da relação com o setor privado é um tanto polêmico, pois envolve posições políticas mais gerais na Fiocruz contrárias a essa aproximação. A questão é que, no caso da genética e das doenças raras, ou você faz um compartilhamento, obviamente respeitando a sua ética, ou vai ficar sem recursos, porque só o financiamento governamental não é suficiente. Essas parcerias deram resultados maravilhosos, como no caso da *Osteogenesis imperfecta* e das mucopolissacaridoses. A gente via as crianças chegando em uma condição muito ruim, resultante de péssima qualidade de vida, e tendo resultados positivos rápidos devido ao medicamento tomado.

Outro momento importante é o desenvolvimento das pesquisas conectando o IFF diretamente às respostas da Fiocruz à epidemia do vírus zika. No contexto epidêmico, a fundação financiou diferentes projetos para investigar o comportamento do vírus, incluindo o projeto “Exposição vertical ao vírus zika e suas consequências no neurodesenvolvimento até os 6 anos de vida: o que sabemos, o que não sabemos e o que precisamos saber”, coordenado pela Maria Elisabeth Lopes Moreira, que também contou com financiamento do Wellcome Trust, da Inglaterra. Esse projeto foi muito importante para a projeção do IFF dentro da Fiocruz e em âmbito internacional. Nosso trabalho com o vírus zika realmente foi um marco, uma vitória para a instituição.

## NOTAS

1 Fernando Olinto formou-se em 1956, pela Faculdade de Ciências Médicas da Universidade do Estado do Rio de Janeiro, e realizou residência em pediatria no Instituto Fernandes Figueira, onde presidiu a Comissão Diretora do Curso de Pediatria e representou a instituição em diferentes fóruns acadêmicos (Gestão..., s.d.).

2 Clóvis Corrêa da Costa formou-se em farmácia na Bahia (1904) e estudou medicina no Rio de Janeiro, formando-se em 1911. Em 1939, passou a dirigir a Maternidade do Instituto Fernandes Figueira, que, mais tarde, passou a chamar-se Maternidade Clovis Corrêa da Costa, a pedido dos médicos do instituto.

3 O IFF/Fiocruz tem em sua estrutura o Centro de Referência Nacional da Rede Global de Bancos de Leite Humano, sendo considerado um Hospital Amigo da Criança pela Organização Mundial da Saúde, pelo Fundo das Nações Unidas para a Infância e pelo Ministério da Saúde.

4 Ludma Dallalana Trota, pediatra com atuação em pneumologia e tisiologia. Pioneira no diagnóstico e no cuidado de pessoas com fibrose cística. Em 1967, organizou um serviço no IFF para realização do teste do suor. Em 1972, criou o primeiro centro brasileiro de referência para o diagnóstico e tratamento da fibrose cística (Sant’Anna, 2020).

5 A abreugrafia é um método de diagnóstico por raio-X desenvolvido pelo médico pneumologista Manuel de Abreu em 1936. Devido ao seu custo baixo e à agilidade de realização do método, a abreugrafia possibilitava realizar radiografias do pulmão que viabilizavam o diagnóstico da tuberculose (Nascimento, 2001).

6 Sergio Arouca, médico e sanitarista, foi professor da Escola Nacional de Saúde Pública na década de 1970. Como presidente da Fiocruz, foi o responsável pela reintegração dos dez cientistas cassados pela ditadura militar. Ocupou a presidência da instituição até abril de 1988, quando se exonerou para concorrer à vice-presidência da República pelo Partido Comunista do Brasil (Sergio Arouca, s.d.).

7 Paulo Roberto Boechat é médico formado pela Universidade Federal Fluminense, com residência em cirurgia pediátrica no IFF. Foi chefe do Departamento de Cirurgia Pediátrica de 1980 a 1985, quando foi eleito diretor do IFF, até 1993. Em 1993-1994, foi *research fellow* da Universidade de Pittsburgh, Pensilvânia. Em 1994, assumiu o cargo de chefe do Departamento de Cirurgia Pediátrica do IFF.

8 O pediatra e sanitarista Paulo Buss foi presidente eleito da Fiocruz durante dois mandatos. Nesse período, atuou como coordenador da Comissão Nacional sobre Determinantes Sociais da Saúde, um fórum estratégico de combate às iniquidades em saúde. Atualmente é presidente da Federação Mundial de Associações de Saúde Pública – uma das mais importantes instituições de saúde pública do mundo.

9 Instituto Refazer é um Grupo de Apoio à Criança e ao Adolescente do Instituto Fernandes Figueira. De acordo com o *site* do instituto, sua missão é “promover a reestruturação de famílias em risco social por meio do PAF – Programa de Atendimento Familiar – de modo a proporcionar a estabilidade da saúde da criança e adolescente, liberando leitos hospitalares da rede SUS, com prioridade para o IFF”.

10 Em 2009, foi instituída a Política Nacional de Atenção Integral em Genética Clínica, que deu ênfase ao aconselhamento genético e às ações no sentido do cuidado integral (Brasil, 20 jan. 2009). Em 2014, o Ministério da Saúde instituiu a Política Nacional de Atenção Integral às Pessoas com Doenças Raras, que teve como destaque a implantação dos Serviços de Atenção Especializada em Doenças Raras (Brasil, 30 jan. 2014).
